# Supplementary material for: Vitamin D Modified DSS-Induced Colitis in Mice via STING Signaling Pathway
Source: Biology (Basel). 2025 Jun 18;14(6):715. doi: 10.3390/biology14060715 (PMC12190092; doi:10.3390/biology14060715)
Supplement: Supplementary file 1 [file biology-14-00715-s001.zip › Supplementary Materials S1: 16s-report of gut microbiota/Groups/diff_analysis/heatmap/KOlogdiffheatmap.pdf]

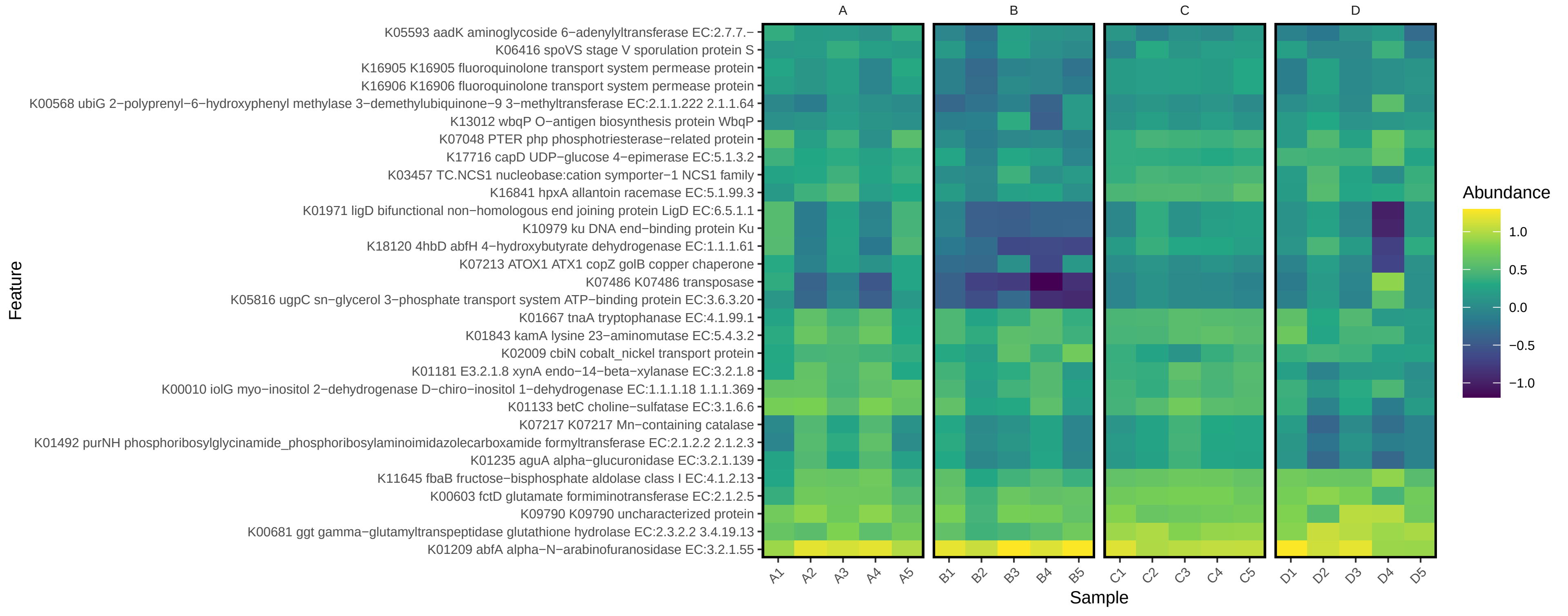
A
B
C
D
K05593 aadK aminoglycoside 6−adenylyltransferase EC:2.7.7.−
K06416 spoVS stage V sporulation protein S
K16905 K16905 fluoroquinolone transport system permease protein
K16906 K16906 fluoroquinolone transport system permease protein
K00568 ubiG 2−polyprenyl−6−hydroxyphenyl methylase 3−demethylubiquinone−9 3−methyltransferase EC:2.1.1.222 2.1.1.64
K13012 wbqP O−antigen biosynthesis protein WbqP
K07048 PTER php phosphotriesterase−related protein
K17716 capD UDP−glucose 4−epimerase EC:5.1.3.2
K03457 TC.NCS1 nucleobase:cation symporter−1 NCS1 family
K16841 hpxA allantoin racemase EC:5.1.99.3
K01971 ligD bifunctional non−homologous end joining protein LigD EC:6.5.1.1
K10979 ku DNA end−binding protein Ku
K18120 4hbD abfH 4−hydroxybutyrate dehydrogenase EC:1.1.1.61
K07213 ATOX1 ATX1 copZ golB copper chaperone
K07486 K07486 transposase
K05816 ugpC sn−glycerol 3−phosphate transport system ATP−binding protein EC:3.6.3.20
K01667 tnaA tryptophanase EC:4.1.99.1
K01843 kamA lysine 23−aminomutase EC:5.4.3.2
K02009 cbiN cobalt_nickel transport protein
K01181 E3.2.1.8 xynA endo−14−beta−xylanase EC:3.2.1.8
K00010 iolG myo−inositol 2−dehydrogenase D−chiro−inositol 1−dehydrogenase EC:1.1.1.18 1.1.1.369
K01133 betC choline−sulfatase EC:3.1.6.6
K07217 K07217 Mn−containing catalase
K01492 purNH phosphoribosylglycinamide_phosphoribosylaminoimidazolecarboxamide formyltransferase EC:2.1.2.2 2.1.2.3
K01235 aguA alpha−glucuronidase EC:3.2.1.139
K11645 fbaB fructose−bisphosphate aldolase class I EC:4.1.2.13
K00603 fctD glutamate formiminotransferase EC:2.1.2.5
K09790 K09790 uncharacterized protein
K00681 ggt gamma−glutamyltranspeptidase glutathione hydrolase EC:2.3.2.2 3.4.19.13
K01209 abfA alpha−N−arabinofuranosidase EC:3.2.1.55
Feature
A1
A2
A3
A4
A5
B1
B2
B3
B4
B5
C1
C2
C3
C4
C5
D1
D2
D3
D4
D5
Sample
Abundance
1.0
0.5
0.0
−0.5
−1.0
